# Microscopical Observation and Transcriptome Analysis Reveal the Effects of High‐Altitude Ecosystem in the Qualities of Different Genetic Varieties *Brassica napus* Resources

**DOI:** 10.1002/ece3.70616

**Published:** 2024-11-21

**Authors:** Zongji Zhang, Xionghua Li, Ri Ming, Yingying Lu, Qinwen Lin, Yafei Yang, Jialin Liao, Yunjuan Li, Lingli Mao, Yang Huang, Li Zhong, Yu Liang

**Affiliations:** ^1^ Guilin Branch of Guangxi Academy of Agricultural Science/Guilin Research Centre of Agricultural Sciences Guilin China; ^2^ Key Laboratory of Ecology of Rare and Endangered Species and Environmental Protection, Guangxi Key Laboratory of Landscape Resources Conservation and Sustainable Utilization in Lijiang River Basin, College of Life Science Guangxi Normal University Guilin China; ^3^ School of Mechanical and Electrical Engineering Guilin University of Electronic Technology Guilin China

**Keywords:** altitude, biomass, bolting, *Brassica napus*, nutritional quality, picking period, transcriptome

## Abstract

Improving the biomass and nutritional quality of *Brassica napus* is important for its breeding and resource conservation, but there are few studies on the effects of high altitude on its biomass and quality. In this study, 27 varieties of *Brassica napus* were cultivated both at 1600 m and 150 m altitudes to investigate the effect of different altitudes in the biomass and quality traits of *B. napus* . At high altitude, all *B. napus* varieties exhibited decreased picking period, reduced fresh and dry weights, diminished stem length and diameter, as well as lowered nitrogen (N), phosphorus (P), and kalium (K) contents. The soluble sugar and cellulose contents of some varieties decreased, while ascorbic acid and protein increased by 74% and 85%, respectively. Furthermore, histology microscopical observation showed cell size increased and cell density decreased in the shoot apical meristem (SAM) after bolting and at high altitude, compared to those cells before bolting and at low altitude. Transcriptome analysis showed that *sucrose phosphate synthase* (*SPS*), *sucrose synthase* (*SUS*)*, fructose‐1,6‐bisphosphate aldolase* (*FBA1*), and *alpha‐galactosidase* (*AGAL2*) genes were significantly up‐regulated during the after‐bolting period at different altitude. This study will be helpful to further understand the influence of high altitude ecosystem on biomass and quality for *Brassica napus* resource and evolution.

## Graphical abstract

Compared with normal low altitude, the high‐altitude ecosystem could change the biomass and quality traits (including the picking period, fresh and dry weights, stem length and stem diameter, the contents of nitrogen, phosphorus, and kalium) of *B. napus* . Transcriptome analysis and microscopical observation of the shoot apical meristem reveal the potential mechanism in high altitude effects. This study will be helpful to further understand the influence of high altitude ecosystem on biomass and quality for *Brassica napus* resource and evolution.

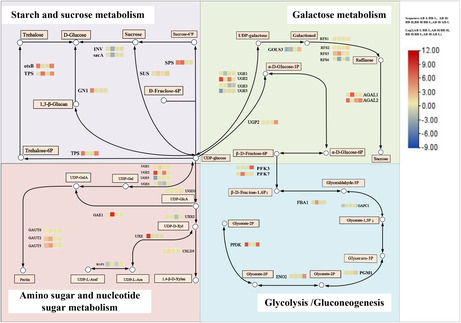

## 1 Introduction

*Brassica napus* (AACC, 2*n* = 38) holds a preeminent position as one of the world's most crucial oil crops (Qian 1984). Its cultivation spans diverse altitudes, indicating a widespread planting area (Wang, Song, et al. 2018). Except for the economic benefit of domestic *B. napus* oil production mode (Zhang et al. 2018), vegetable use of *B. napus* is an effective way to extend the industrial chain and economic benefits. This vegetable application serves as a valuable addition to the crop's production mode. Previous research has highlighted the significant influence of soil, light, sowing time, fertilization, and altitude on the yield, biomass, and quality of *B. napus* (Cong, Zhang, and Lu 2019; Gu et al. 2023; Hu et al. 2023; Wei et al. 2022). Altitude is an important factor affecting oil content and agronomic traits of *B. napus* seeds (Yang et al. 2021a). Different altitudes have different climatic conditions, light intensity, and soil characteristics (Pan et al. 2009), which may affect the biomass and quality of *B. napus* . With the global climate change and the development of high‐mountain agriculture, high‐altitude regions are gradually emerging as crucial areas for crop cultivation (Maharjan et al. 2023). *Brassica napus* , one of the globally significant oilseed crops, holds great importance for ensuring edible oil supply and promoting sustainable agricultural development in high‐altitude areas (Yang et al. 2024). However, the current lack of research on the growth characteristics and nutritional quality changes of *B. napus* under high‐altitude conditions has constrained its widespread cultivation and efficient utilization in these regions. Therefore, investigating the effects of high‐altitude environments on the biomass and quality formation of different *B. napus* varieties is very important.

At present, the researches on the biomass and quality control of vegetable use *B. napus* mainly focus on the picking period (Sun et al. 2021), picking times (Zhao et al. 2023), and variety selection (Liu et al. 2022). There are few researches on the mechanism of altitude affecting the picking period, picking times, biomass, and quality formation of various types of *B. napus* . The impacts of high‐altitude environmental conditions on plants are multifaceted, including changes in growth characteristics (Li et al. 2016), physiological and biochemical responses (Zhu et al. 2013), alterations in anatomical structure (He, Wu, and Jia 2007), and adaptive strategies (Zhang et al. 2023a). In other plants, studies showed that the altitude affects the plant growth and biomass. For example, the plant height, leaf area, stem length, and fresh weight of *Panicum antidotale* exhibit a significant decreasing trend with increasing altitude, while the intercellular spaces in the mesophyll cells enlarge (Irshad et al. 2024). As elevation increases, the aboveground biomass of the two sorghum species decreases, whereas root growth increases (Ahmad et al. 2022). As increase in altitude, the rates of photosynthesis, respiration, and water use efficiency in plants may undergo changes to adapt to unfavorable conditions such as low temperature and hypoxia (Ahmad et al. 2018; Iqbal et al. 2024). Other studies have shown that under the stress of high altitude and low temperature, the accumulation of ascorbic acid in plant cells increases, which can prevent the harmful effects of reactive oxygen species (ROS) (Kumar et al. 2015). High altitude is conducive to the accumulation of protein, amino acids, and other substances, but it is not conducive to the accumulation of fiber substances (Wang et al. 2017). Although some studies have explored the effects of altitude on plant growth and development, research specifically focused on *B. napus* remains inadequate, particularly regarding the changes in its biomass and nutritional quality under different altitudinal conditions. It is significant to understand how high‐altitude environmental factors (such as light and temperature) influence biomass accumulation, nutrient synthesis, and the underlying molecular mechanisms in *B. napus* . Therefore, this study aims to investigate the potential effects of different altitudes on the growth mechanism of *B. napus* , which can provide support for breeding to improve its yield and quality.

To investigate the impact of altitude on the biomass, picking period, nutritional quality, and nutrient composition of *B. napus* , a comparative analysis was conducted on the key characteristics of the crop at varying altitudes. The findings revealed a decrease in biomass, soluble sugar, and cellulose content as altitude increased, whereas ascorbic acid and protein levels exhibited an upward trend. Additionally, nitrogen, phosphorus, and kalium concentrations in *B. napus* decreased with increasing altitude. Notably, significant differences were observed in the cultivars ZYCT02 and Huayouza 655R under these parameters. Microscopic observations further showed enlarged and more loosely packed cells in the shoot apical meristem (SAM) after bolting (AB) and at higher altitudes, compared to pre‐bolting and lower altitudes. Transcriptome analysis of Huayouza 655R revealed that *SPS*, *SUS*, *FBA1*, and *AGAL2* genes play a pivotal role in regulating metabolism in vegetable‐oriented *B. napus* . This study sheds light on the varying trends in traits, biomass, and quality of *B. napus* at different altitudes, providing a scientific foundation for the cultivation of vegetable‐oriented *B. napus* and the optimization of agricultural management practices.

## 2 Materials and Methods

### 2.1 Plant Materials and Growing Condition

**FIGURE 1:**
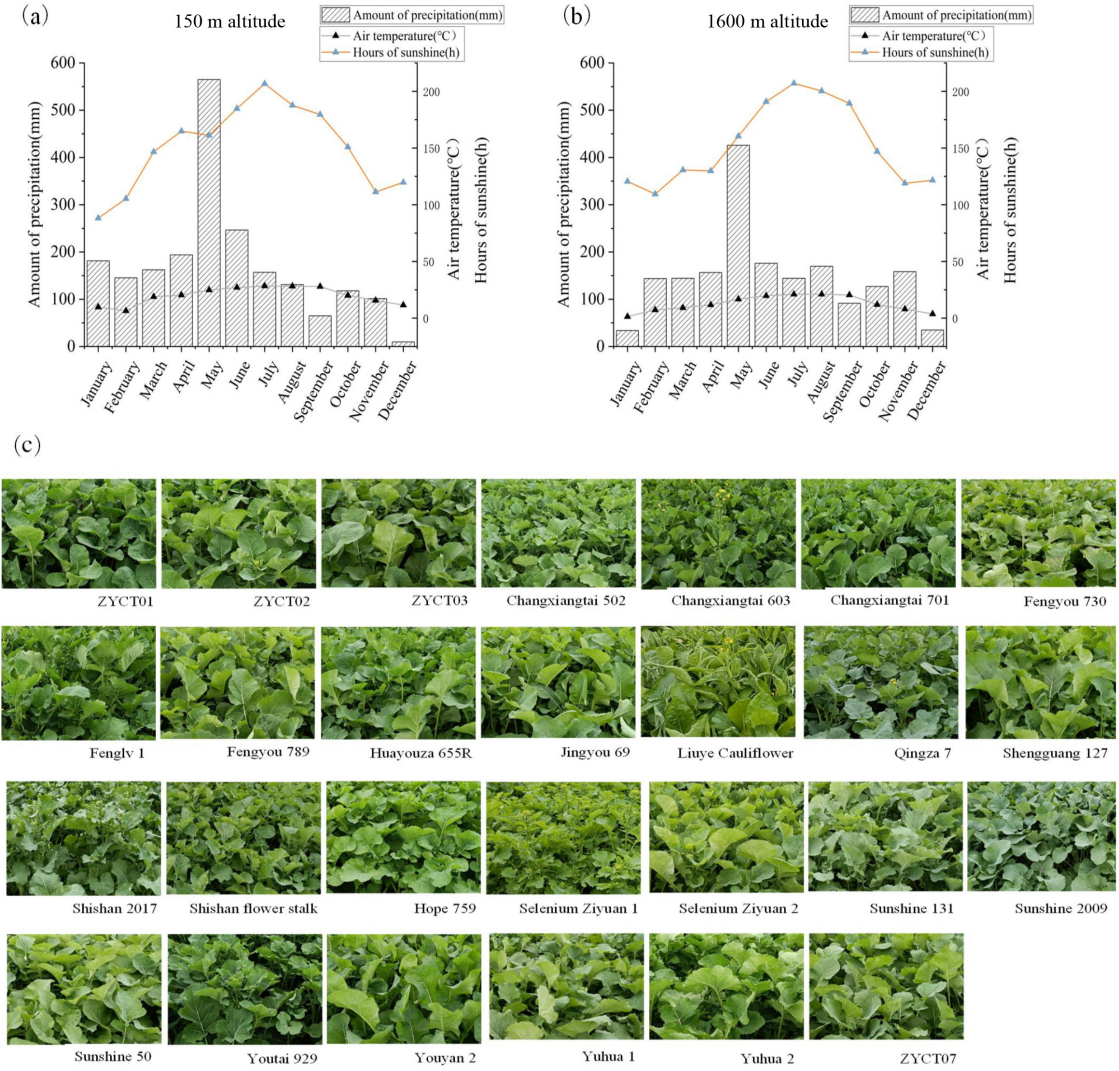
Growing environment and condition of *B. napus* . (a and b) The average annual rainfall, average annual temperature, and average annual sunshine hours at 150 and 1600 m altitudes. (c) Growth patterns of *B. napus* grown at 150 m altitude.

A total of 27 varieties of *B. napus* were used in this study, including ZYCT01, ZYCT02, ZYCT03, Changxiangtai 502, Changxiangtai 603, Changxiangtai 701, Fengyou 730, Fenglv 1, Fengyou 789, Huayouza 655R, Jingyou 69, Liuye Cauliflower, Qingza 7, Shengguang 127, Shishan 2017, Shishan flower stalk, Hope 759, Selenium Ziyuan 1, Selenium Ziyuan 2, Sunshine 131, Sunshine 2009, Sunshine 50, Youtai 929, Youyan 2, Yuhua 1, Yuhua 2, Yuhua 7. Importantly, all these 27 varieties were cultivated at both 150 m and 1600 m altitudes to assess their growth in different elevations. During the planting period, the average annual rainfall, average annual temperature, and average annual sunshine hours at 150 m altitude are 172.96 mm, 19.93°C, and 150.56 h, respectively. The average annual rainfall, average annual temperature, and average annual sunshine hours at 1600 m altitude are 150.45 mm, 12.70°C, and 152.15 h respectively (Figure 1a,b). Although the meteorological conditions are slightly different at high and low altitudes, *B. napus* can grow normally (Figure 1c; Figure S1).

The experiment was set up in two altitude areas: 150 and 1600 m. The row spacing of each plant was 16 × 30 cm, and the seedlings were thinned and even during the whole growth period. Urea was applied at a rate of 150 kg/hm^−2^ after the first harvest of each *B. napus* variety. Growth, total biomass, and quality‐related indexes were recorded during the planting process.

### 2.2 Measurement of Picking Periods, Fresh Weight, Dry Weight, Ascorbic Acid, Soluble Sugar, Cellulose, Protein, and Nitrogen, Phosphorus, and Kalium Contents of *B. napus*

The time between the start of *B. napus* picking and the final picking is referred as a complete picking period. The water and soil were removed from the plants, the fresh weight of each plant was weighed and recorded, and the sampling was repeated three times for each treatment group. The previously measured fresh weight of *B. napus* plants were labeled, placed in a drying oven, and dried at 80°C for 48 h. Each *B. napus* plant was weighed after it cooled to room temperature. The value of each weighing was recorded and the average value was calculated as the fresh and dry weight of *B. napus* for that period (Alam et al. 2014).

The protein of *B. napus* was determined by Coomassie brilliant blue method (Ågren 2008). Determination of ascorbic acid in *B. napus* by iron ion REDOX method (Ågren 2008). The cellulose and soluble sugar of *B. napus* were determined by anthrone method (Ågren 2008).

After digestion with concentrated H_2_SO_4_–H_2_O_2_, the nitrogen and potassium of plants were determined by flow injection analyzer, and kalium was determined by flame spectrophotometry (Ma 2023).

### 2.3 Microscopic Horizontal Section Observation of *B. napus* SAM Cells

Significant differences in picking period, N, P, and K contents, morphological indexes, nutritional quality, and biomass of Huayouza 655R were found between high and low altitudes (1600 and 150 m). SAM of *B. napus* variety Huayouza 655R were collected [before and after bolting at low altitude (BB‐L and AB‐L), and before and after bolting at high altitude (BB‐H and AB‐H), respectively]. Three biological replicates were selected and stored in 50%–70% FAA (formaldehyde‐acetic acid) and 2.5% glutaraldehyde fixatives, respectively (Buszewska‐Forajta et al. 2019). The SAM was subsequently prepared for scanning electron microscopy (SEM) observation and the production of paraffin sections for observation. The modified paraffin sectioning method (Li 1987) was employed to create the sections. 3–5 mm of SAM from the FAA fixative was dehydrated with an alcohol gradient and then treated with xylene clear. The SAM were then embedded in wax and sliced using a paraffin sectioning machine. The section thickness was set at 4 μm. Safranin‐fast green double staining was used, and the sections were sealed with neutral gum. The SEM slicing technique was utilized in this study. The meristem tissue from the SAM of rapeseed plants was excised into cubes of approximately 1 mm and fixed in 2.5% glutaraldehyde at 4°C for 2 h (Chen et al. 2019). After this, the tissue samples were dehydrated in a graded ethanol series, including 30%, 50%, and 70% ethanol solutions, and subsequently 3–5 rounds of dehydration were undergone by using absolute ethanol (Chen et al. 2019). Following drying, gold sputtering was applied to the samples, and observation and photography were carried out using the JSM6360LV scanning electron microscope at a voltage of 10 kV (Chen et al. 2019).

### 2.4 Transcriptome Sequencing, and Library Construction

Total RNA was extracted from apical meristem samples and assayed for concentration and purity using Nanodrop2000. The integrity of the RNA was determined by agarose gel electrophoresis, and the RIN value was determined using an Agilent 2100 Bioanalyzer system. Sequencing experiments were performed using the Illumina TruseqTM RNA sample prep Kit method for library construction (Huan et al. 2022). Reads were aligned to the published *B. napus* reference genome sequence using TopHat (Trapnell et al. 2010) (http://cbi.hzau.edu.cn/cgi‐bin/rape/download_ext).

### 2.5 Transcriptome Analysis

To comprehensively obtain the gene function information of oilseed rape for better study of its function, the assembled gene sequences were matched to the following six major databases in this study: KEGG, GO, Pfam, NR, Swiss‐prot, EggNOG. The data obtained in the analysis of gene expression level were analyzed by DESeq2 software (Love, Huber, and Anders 2014). Using sequencing data with an error rate below 0.1%, we screened significantly differentially expressed genes by applying the criteria of |log2FC| > 1 and padjust < 0.05. To further investigate the functions of the differentially expressed genes, the GO and KEGG databases were used for pathway enrichment analyses as well.

### 2.6 Statistical Analysis

Microsoft Excel 2010 software was used to process the data. Mapping with Origin2021 software; SPSS 26.0 analysis software was used for ANOVA statistical analysis and correlation analysis, and Duncan's test showed the significance of the difference. Differences at *p* < 0.05 were considered significant (Mishra et al. 2019).

## 3 Results

### 3.1 Altitude‐Related Differences in Picking Period of Vegetable‐Use *B. napus*

**FIGURE 2:**
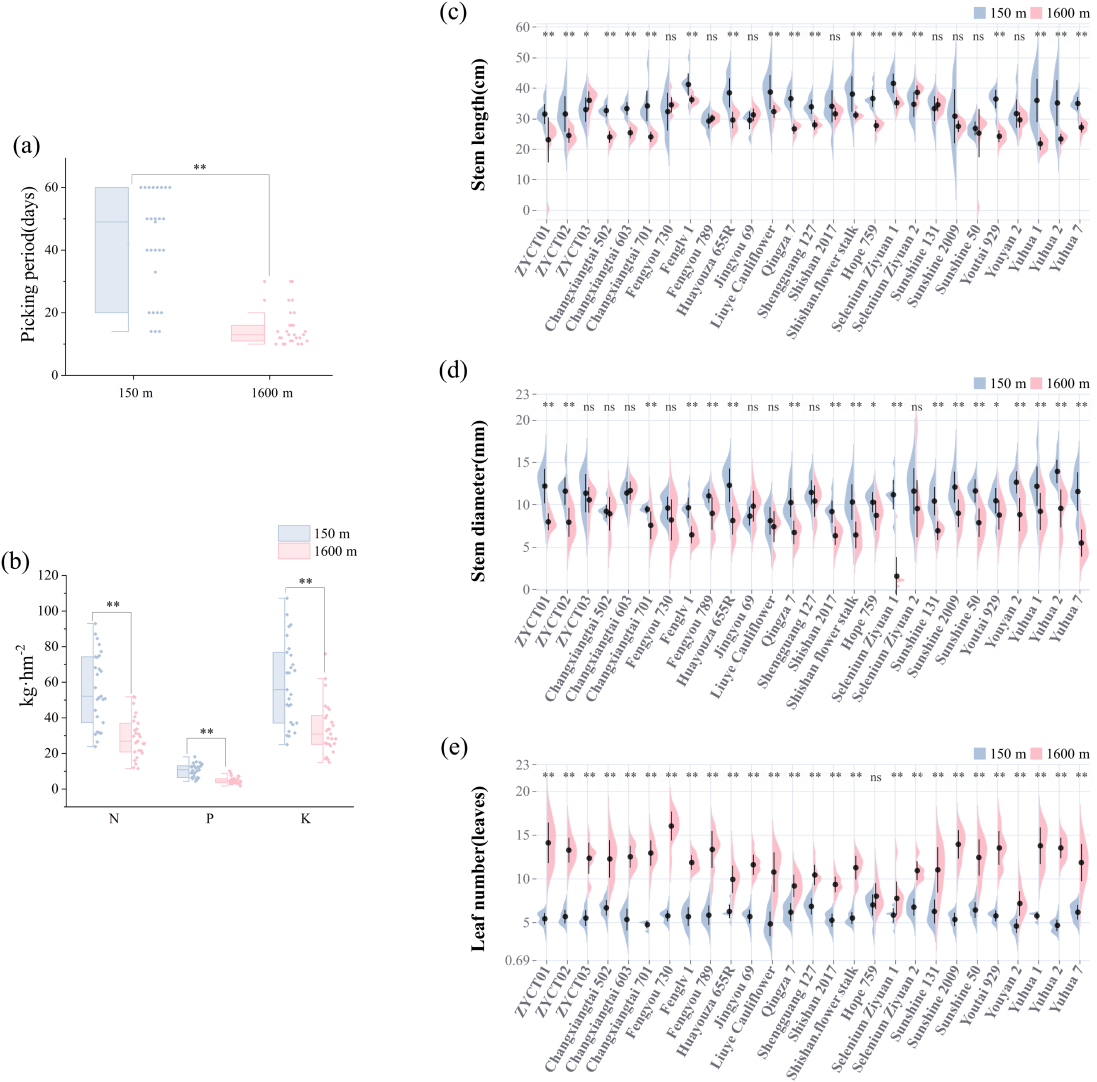
Picking period, nutrients, and morphology of *B. napus* . (a) Picking period of *B. napus* at 150 m altitude and 1600 m altitude. (b) Nutrients (N, P, and K) in *B. napus* at 150 m altitude and 1600 m altitude. (c–e) Stem length, stem diameter, and leaf number of *B. napus* at 150 m altitude and 1600 m altitude. ** indicate a *p*‐value less than 0.01.

To study the supply period of *B. napus* at different altitudes, the picking period of *B. napus* planted at different altitudes was calculated. Under different altitude treatment, the picking period of *B. napus* in 150 m altitude area was significantly longer than that in 1600 m altitude area. The average picking period of *B. napus* in the 150 m altitude area was 42 days, which is 36% longer compared to the picking period in the 1600 m altitude area (Figure 2a). In general, with the increase in altitude, the picking period of *B. napus* becomes shorter. These results suggest that the growth rate of *B. napus* accelerates with increasing altitude, leading to its earlier maturity and harvestability stage. The large temperature difference between day and night and the long sunshine time at high altitude may have acted together on *B. napus* , leading to its earlier maturity and shorter growth cycle (Table S1).

### 3.2 Altitude Effects on Nitrogen, Phosphorus, and Kalium Contents in Vegetable‐Use *B. napus*

The improvement of crop agronomic traits is closely related to nutrient uptake and utilization. To understand the nutrient absorption and utilization of *B. napus* at different altitudes, the contents of nitrogen (N), phosphorus (P), and kalium (K) in *B. napus* were measured. The contents of N, P, and K in *B. napus* at 1600 m altitude were significantly lower than that at 150 m altitude (Figure 2b). At 150 m altitude, the contents of N, P, and K in *B. napus* were 56.41 kg·hm^−2^, 10.55 kg·hm^−2^, and 59.33 kg·hm^−2^, respectively. At 1600 m altitude, the contents of N, P, and K in *B. napus* were 29.07 kg·hm^−2^, 4.98 kg·hm^−2^, and 34.00 kg·hm^−2^, respectively (Table S2). Soil N, P, and K contents were lower at the high altitude of 1600 m than at the low altitude of 150 m (Table S3). This may be due to the lower temperatures and barometric pressure at high altitudes, which affects the activity of microorganisms in the soil. Low temperatures may limit microbial activity, resulting in slower release and lower levels of nutrients such as N, P, and K in the soil.

### 3.3 Altitude‐Dependent Variations in *B. napus* Stem and Leaf Traits

Stem length, stem diameter, and leaves were also measured to compare the basic indexes of plant morphology at different altitudes. The stem lengths of the following 18 varieties were significantly reduced at an altitude of 1600 m compared to their stem lengths at 150 m: ZYCT01, ZYCT02, ZYCT03, Changxiangtai 502, Changxiangtai 603, Changxiangtai 701, Fenglv 1, Huayouza 655R, Liuye Cauliflower, Qingza 7, Shengguang 127, Shishan flower stalk, Hope 759, Selenium Ziyuan 1, Youtai 929, Yuhua 1, Yuhua 2, and Yuhua 7 (Figure 2c). Specifically, at 150 m, Selenium Ziyuan 1 exhibited the longest stem length (41.50 cm), whereas Sunshine 50 had the shortest (26.75 cm). At 1600 m, Selenium Ziyuan 2 had the longest stem length (38.57 cm) and Yuhua 1 had the shortest stem length (21.74 cm).

The stem diameters of 19 *B. napus* cultivars were notably reduced at an altitude of 1600 m compared to those at 150 m (Figure 2d). At the lower altitude, Yuhua 2 exhibited the largest stem diameter (13.92 mm), whereas Liuye Cauliflower had the smallest (8.09 mm). At 1600 m, Changxiangtai 603 had the largest stem diameter (11.65 mm) and Selenium Ziyuan 1 had the smallest stem diameter (1.57 mm).

*Brassica napus* plants at 1600 m altitude had significantly more leaves than those at 150 m (Figure 2e). At 150 m, four varieties (Changxiangtai 502, Shengguang 127, Hope 759, Selenium Ziyuan 2) had the maximum of seven leaves, while eight varieties (ZYCT01, Changxiangtai 603, Changxiangtai 701, Liuye Cauliflower, Shishan 2017, Sunshine 2009, Youyan 2, Yuhua 2) had the minimum of five leaves. At 1600 m, Fengyou 730 had the most leaves (16), and Youyan 2 had the least leaves (seven). In general, vegetable use *B. napus* grown at high altitude adapts to its environment through morphological changes.

### 3.4 Comparative Analysis of Soluble Sugar and Cellulose Contents in Vegetable‐Use *B. napus* at High and Low Altitudes

**FIGURE 3:**
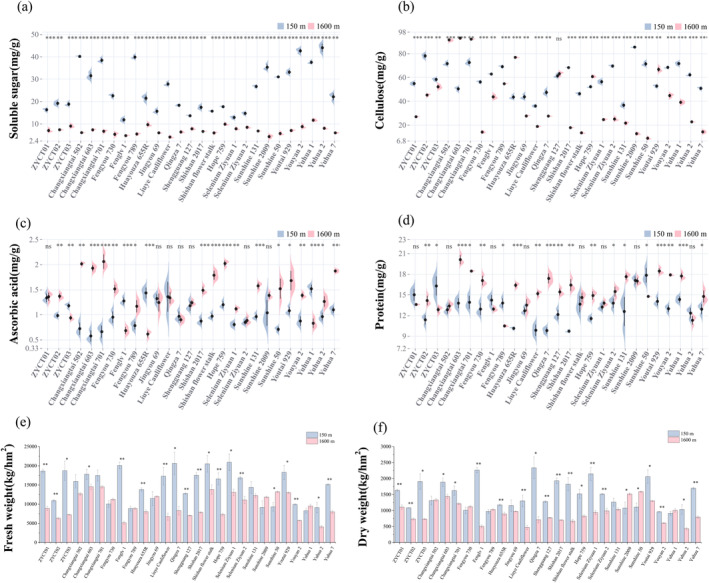
Nutritional quality of *B. napus* . (a–f) Soluble sugars, cellulose, ascorbic acid, protein, fresh weight, and dry weight contents of *B. napus* at 150 m altitude and 1600 m altitude.

To investigate the variations in the nutritional quality of *B. napus* at different altitudes, the analysis of the soluble sugar and cellulose contents of plants cultivated at two distinct altitudes was performed. The findings uncovered significant differences in nutritional qualities among varieties at these altitudes. The soluble sugar content of all *B. napus* varieties at 1600 m altitude was significantly lower than that at 150 m altitude (Figure 3a). At 150 m altitude, Yuhua 2 had the highest soluble sugar content (44.01 mg/g). This high content could be linked to their optimal growing conditions at low altitude, favoring sugar production and accumulation. While Fenglu 1 had the lowest soluble sugar content (11.80 mg/g). Shifting to the 1600 m altitude, Yuhua 1 emerged as the variety with the highest soluble sugar content (11.62 mg/g), even if this is still much lower than at 150 m. While Liuye Cauliflower has the lowest soluble sugar content (4.07 mg/g), indicating a significant reduction in sugar accumulation under high altitude conditions (Table S4).

The cellulose contents of Changxiangtai 502, Changxiangtai 603, Changxiangtai 701, Huayouza 655R, Hope 759, Youtai 929 at 1600 m altitude are significantly higher than that at 150 m altitude. However, the cellulose contents of 20 *B. napus* varieties were notably reduced at an altitude of 1600 m compared to those at 150 m (Figure 3b). This suggests that while some varieties responded to the higher altitude by increasing cellulose content, the general trend was a decreased cellulose content. Specifically, at 150 m altitude, Sunshine 2009 exhibited the highest cellulose content (85.43 mg/g), whereas Liuye Cauliflower demonstrated the lowest (36.00 mg/g). At 1600 m altitude, Changxiangtai 603 had the highest cellulose content (92.99 mg/g) and the Sunshine 50 had the lowest cellulose content (8.97 mg/g) (Table S5). These results indicate that high‐altitude planting has a complex impact on the accumulation of soluble sugars and cellulose in *B. napus* . It not only leads to a significant decrease in soluble sugar and cellulose content in certain varieties but also causes an increase in cellulose content in some varieties. This variation reflects the diverse adaptation strategies of plants in response to different environmental pressures and significantly affects the nutritional quality of *B. napus* .

### 3.5 Comparative Study of Ascorbic Acid and Protein Contents in Vegetable‐Use *B. napus* at Different Altitudes

Similarly, we have analyzed ascorbic acid and protein in *B. napus* grown at different altitudes. At 1600 m altitude, the ascorbic acid contents of 16 *B. napus* varieties, such as ZYCT02, Changxiangtai 502, and so on, are significantly higher compared to those grew at 150 m. The ascorbic acid levels in these varieties have significantly risen, suggesting enhanced production or accumulation of this essential vitamin at higher altitudes. Conversely, varieties like ZYCT03, Fenglv 1, and a few more show a significant decrease in ascorbic acid content at this higher altitude (Figure 3c). At 150 m altitude, Yuhua 1 had the highest ascorbic acid content (1.52 mg/g), and Changxiangtai 603 had the lowest ascorbic acid content (0.57 mg/g). At 1600 m altitude, the ascorbic acid content of Changxiangtai 701 was the highest (2.06 mg/g), and the ascorbic acid content of Huayouza 655R was the lowest (0.61 mg/g) (Table S6). These results indicate that altitude has a significant impact on the ascorbic acid content of *B. napus* , with specific varieties showing distinct patterns of changes.

The protein contents of most *B. napus* varieties were significantly higher at 1600 m than at 150 m altitude (Figure 3d), except for ZYCT03, Fengyou 789, and Sunshine 50, which showed lower protein contents at 1600 m. At 150 m, Sunshine 2009 had the highest protein content (17.08 mg/g), and Shishan 2017 had the lowest protein content (9.71 mg/g). At 1600 m, Changxiangtai 603 had the highest protein content (20.10 mg/g), which was notably higher than the maximum value observed at 150 m. Fengyou 789 showed the lowest protein content (11.47 mg/g) at 1600 m (Table S7). While most varieties experienced an increasing protein content in high altitude, indicating enhanced protein synthesis and accumulation, a few varieties exhibited a decrease. This divergence may be caused by the differences in their adaptability to the environmental stresses associated with high altitude. Overall, high‐altitude cultivation increases the ascorbic acid and protein content of *B. napus* , thereby affecting its nutritional quality.

### 3.6 Differences in Fresh and Dry Weight of Vegetable‐Use *B. napus* at Varying Altitudes

The aboveground biomass is one of the important parameters to judge the growth state of *B. napus* . The measurement of fresh and dry weights of *B. napus* growth at different altitudes was performed. Most varieties had significantly lower fresh and dry weights at 1600 m compared to 150 m, except for eight varieties (Changxiangtai 502, Changxiangtai 701, Fengyou 730, Fengyou 789, Jingyou 69, Sunshine 131, Sunshine 2009, Yuhua 1). At 150 m, Selenium Ziyuan 1 had the highest fresh weight (20925.04 kg·hm^−2^) and Qingza 7 had the highest dry weight (2339.28 kg·hm^−2^). At 1600 m, Changxiangtai 603 had the highest fresh weight (14543.38 kg·hm^−2^), and Sunshine 50 had the highest dry weight (1593.05 kg·hm^−2^). In general, high altitudes reduce biomass by weakening the fresh weight and dry weight of *B. napus* .

### 3.7 Altitude‐Driven Transformations of SAM Cell Morphology in Vegetable‐Use *B. napus*

**FIGURE 4:**
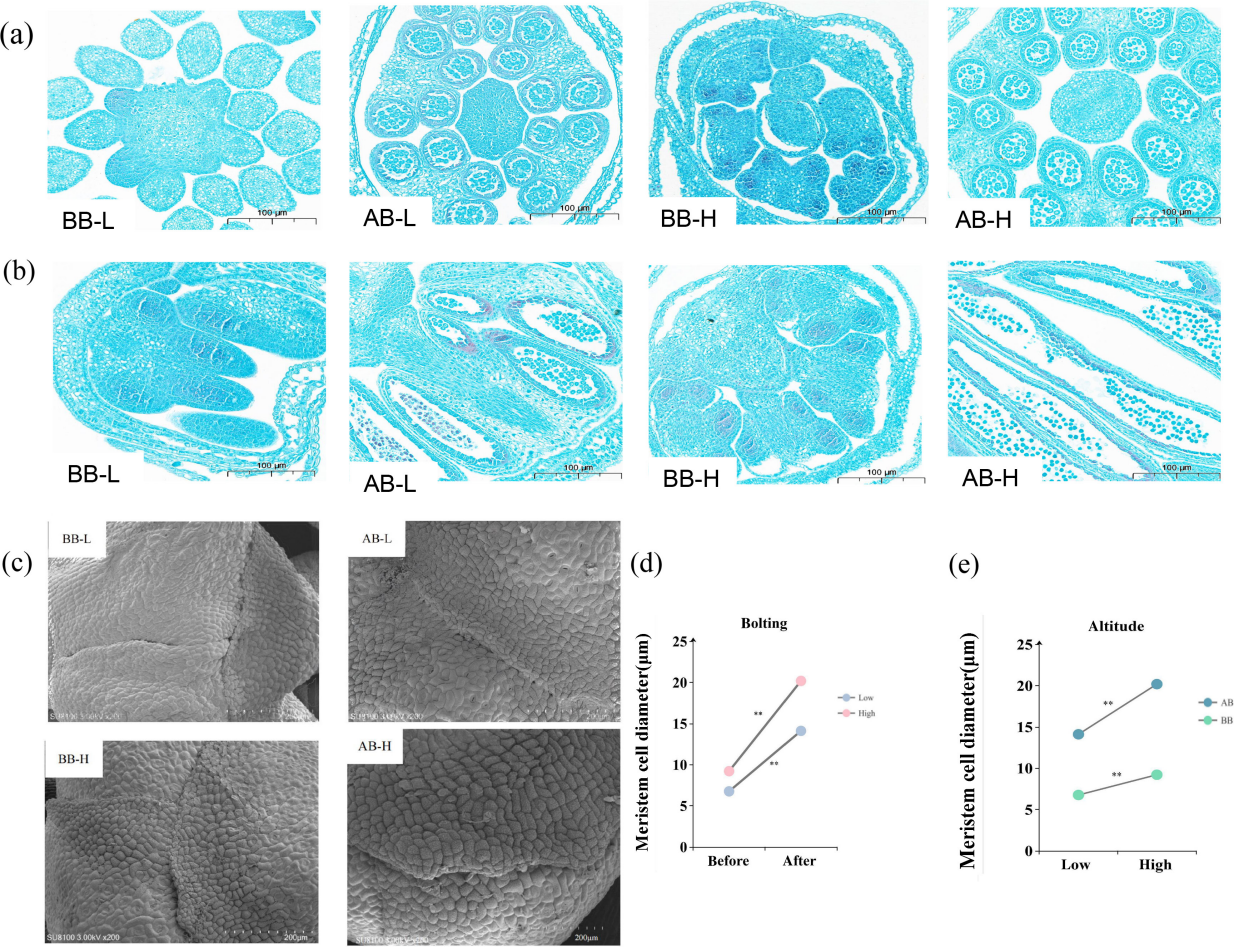
Cell morphology of SAM. (a) Transverse paraffin sections of SAM of Huayouza 655R from BB‐L, AB‐L, BB‐H, and AB‐H. (b) Longitudinal paraffin sections of SAM of Huayouza 655R from BB‐L, AB‐L, BB‐H, and AB‐H. (c) Scanning electron microscope images of SAM tissue of Huayouza 655R from BB‐L, AB‐L, BB‐H, and AB‐H. (d) Diameter of the meristematic tissue cells before and after bolting. (e) Diameter of the SAM cells at high and low altitude.

We used Huayouza 655R, which has notable variations in multiple metrics, as the subject to explore the effects of high altitude on *B. napus* growth. Transverse and longitudinal paraffin sections and electron microscope scans (SEM) of SAM were prepared for observation (Figure 4a–c). Results showed that SAM cells underwent changes in size and density during the transition from the BB to the AB. After bolting at low altitude (AB‐L), the cells increased in size by 49% and were more loosely packed than BB at low altitude (BB‐L) (Figure 4d). Similarly, AB at high altitude (AB‐H), the cells were larger and more dispersed than BB at high altitude (BB‐H), showing an increase of over 46%. BB‐H cells were 73% larger and more loose than BB‐L (Figure 4e). Likewise, AB‐H cells were 69% larger and more dispersed than AB‐L (Figure 4e). These results suggest that high‐altitude cultivation reduces the ability of meristematic tissue differentiation in *B. napus* .

### 3.8 Transcriptome Analysis Unveils Regulation Mechanisms of Vegetable‐Use *B. napus* Across Growth Periods and Altitudes

**FIGURE 5:**
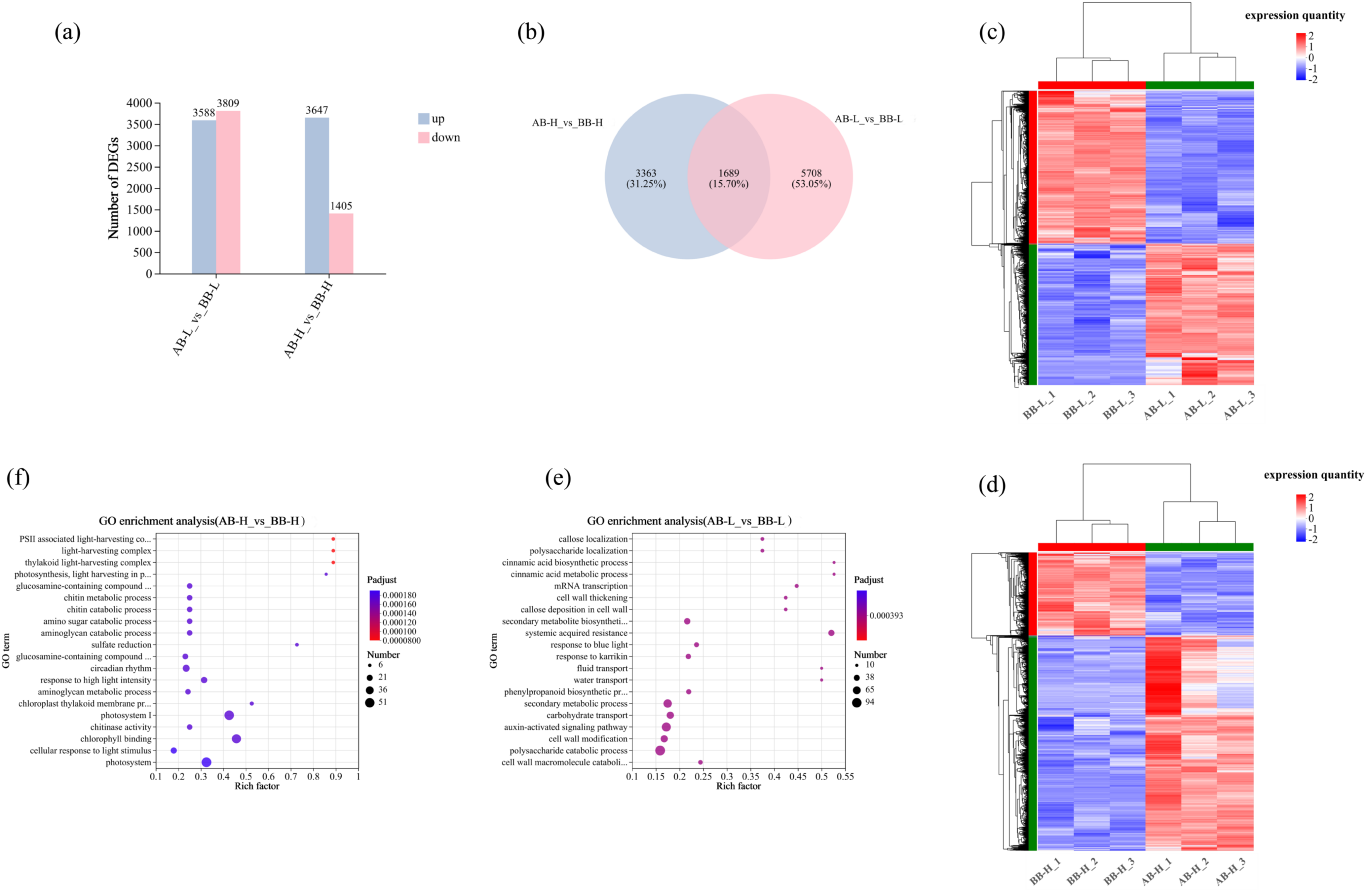
Transcriptome results of *B. napus* under different growth periods. (a) AB‐L vs. BB‐L and AB‐H vs. BB‐H of DEGs. The horizontal coordinates represent the different groups of differential comparisons and the vertical coordinates represent the corresponding number of up‐ and down‐regulated genes. |log_2_FC| ≥ 2 and padjust < 0.05. (b) Venn diagram of differentially expressed gene sets AB‐L vs. BB‐L and AB‐H vs. BB‐H before and after bolting in *Brassica napus* under high‐ and low‐altitude conditions. Different‐colored circles denote distinct gene sets. Numbers within each circle sum up the total genes, while overlaps show shared genes. (c and d) Hierarchical clustering analysis heatmap of differentially expressed gene sets AB‐L vs. BB‐L and AB‐H vs. BB‐H before and after bolting under high‐ and low‐altitude conditions. Generate a heatmap using hierarchical clustering algorithm with RSEM software. In the figure, columns represent samples and rows represent genes. Colors indicate normalized gene expression levels, with red denoting higher expression and blue denoting lower expression. The gene clustering dendrogram on the left illustrates expression similarity, while the sample clustering dendrogram above reveals the closeness of gene expression patterns among samples. (e and f) GO enrichment bubble chart comparing differentially expressed gene sets AB‐L vs. BB‐L and AB‐H vs. BB‐H before and after bolting in *Brassica napus* . The bubble chart uses BH's multiple testing correction method. Rich factor represents the ratio of the enriched sample number to the background number in this GO term. The larger the Rich factor, the greater the degree of enrichment. The size of the dots indicates the number of genes in this GO term, and the color of the dots corresponds to different Padjust ranges.

We analyzed the transcriptome of the SAM in *B. napus* to explore changes in nutrient content and quality during various growth periods. At both high and low altitudes, a significant number of genes were up‐ and down‐regulated AB compared to the material that were BB, indicating that bolting can markedly alter the gene expression in *B. napus* (Figure 5a). Venn diagram analysis identified 1689 differentially expressed genes (DEGs) between two gene sets, representing 15.70% of total DEGs (Figure 5b). Heatmaps, generated via hierarchical clustering, revealed two distinct gene clusters (Figure 5c,d). At the same altitude, significant differences in gene expression emerged BB and AB, indicating a remarkable effect of this bolting process on the growth, development, and quality of *B. napus* . To identify the biological processes regulated by DEGs in *B. napus* BB and AB, GO enrichment analysis was conducted via sequence enrichment (Figure 5e,f). The results indicated that at low altitude, genes were predominantly enriched in sugar metabolism, growth hormone signaling, polysaccharide catabolism, cell wall modification, and carbohydrate transport pathways AB (Figure 5e). At high altitude, the enrichment AB was notable in genes related to the photosystem and chlorophyll synthesis, primarily involved in photosynthesis metabolism (Figure 5f).

**FIGURE 6:**
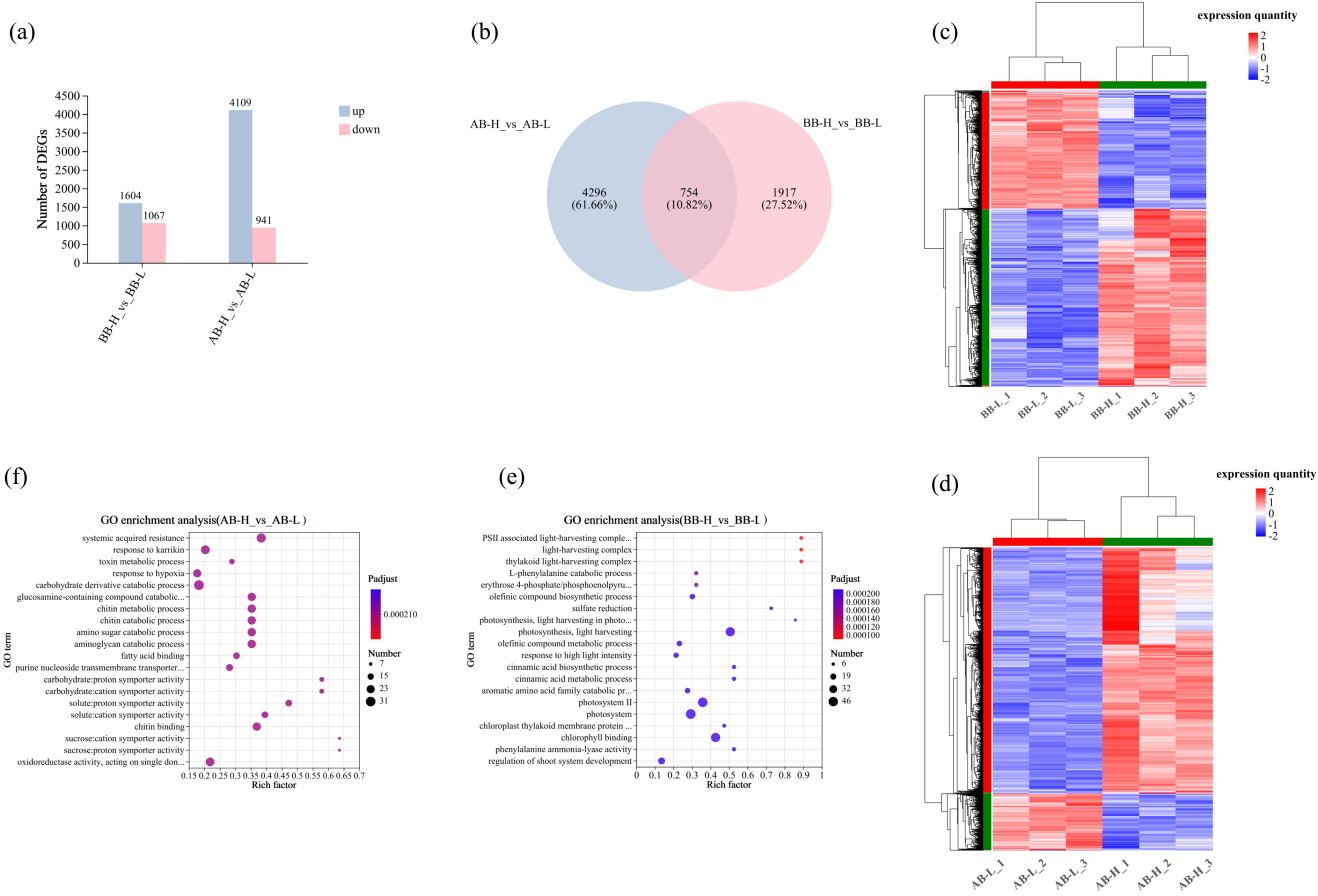
Transcriptome results of *B. napus* at different altitudes. (a) BB‐H vs. BB‐L and AB‐H vs. AB‐L of DEGs. The horizontal coordinates represent the different groups of differential comparisons and the vertical coordinates represent the corresponding number of up‐ and down‐regulated genes. |log_2_FC| ≥ 2 and padjust < 0.05. (b) Venn diagram of differentially expressed gene sets BB‐H vs. BB‐L and AB‐H vs. AB‐L before and after bolting in *Brassica napus* under high‐ and low‐altitude conditions. Different‐colored circles denote distinct gene sets. Numbers within each circle sum up the total genes, while overlaps show shared genes. (c and d) Hierarchical clustering analysis heatmap of differentially expressed gene sets BB‐H vs. BB‐L and AB‐H vs. AB‐L before and after bolting under high‐ and low‐altitude conditions. Generate a heatmap using hierarchical clustering algorithm with RSEM software. The annotations for the heatmap are the same as those for the heatmap in Figure 5. (e and f) GO enrichment bubble chart comparing differentially expressed gene sets BB‐H vs. BB‐L and AB‐H vs. AB‐L at high and low altitudes. The bubble chart uses BH's multiple testing correction method. The annotations for the bubble chart are the same as those for the bubble chart in Figure 5.

Transcriptome analysis was performed on the SAM of *B. napus* to investigate the effects of different altitudes on the growth, development and nutritional quality of *B. napus* . Compared to low altitudes, a significantly greater number of gene expressions were regulated at high altitudes BB and AB, indicating that an increase in altitude can significantly alter the gene expression in *B. napus* during the bolting process (Figure 6a). Venn diagram analysis showed that there were 754 differential genes between the two gene sets, accounting for 10.82% of the total number of DEGs (Figure 6b). To determine the biological processes regulated by DEGs in *B. napus* at varying altitudes, we performed GO enrichment analysis. Before bolting, genes at high altitudes were enriched in photosynthesis‐related processes like the light‐trapping complex (Figure 6e). After bolting, high altitudes showed enrichment in sugar metabolism‐related genes (Figure 6f).

**FIGURE 7:**
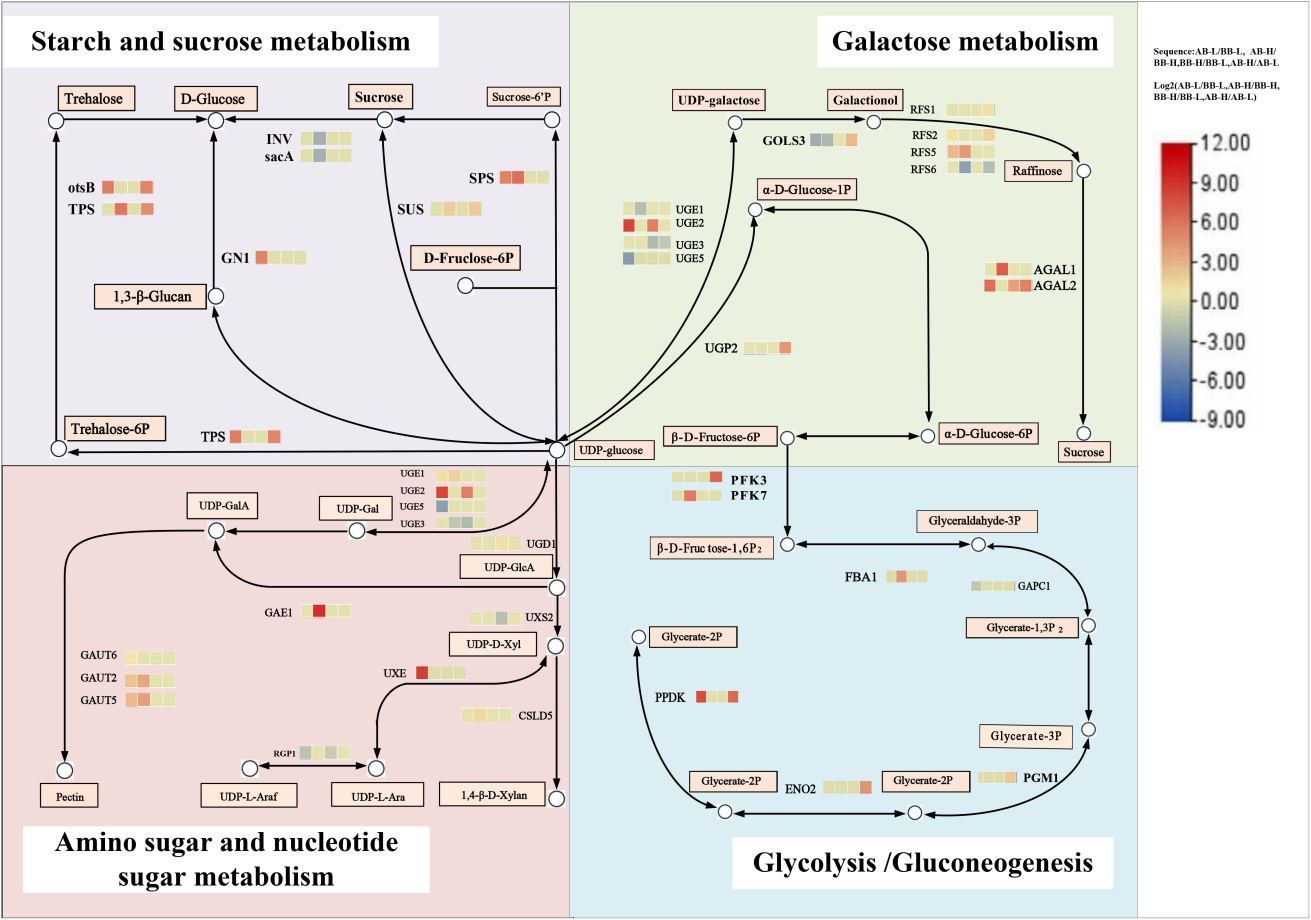
Key metabolic pathways in *B. napus* at different altitudes and during different growth periods. Based on the analysis of transcriptome data, the KEGG database was utilized to search for differentially expressed genes. Pathways enriched with the majority of these genes were then identified, enabling the construction of a KEGG pathway diagram focusing on key differentially expressed genes. This approach provides a comprehensive visualization of the critical metabolic and signaling pathways involved in the transcriptional response. Red indicates up‐regulation, blue indicates down‐regulation.

Enrichment analyses of DEGs and mappings of KEGG pathways show the effects of varying altitudes and growth periods on *B. napus* (Figure 7, Tables [Link], [Link], [Link], [Link]). KEGG enrichment analysis revealed significant up‐regulation of starch and sucrose metabolism genes and down‐regulation of glycolysis or gluconeogenesis genes in *B. napus* at low altitude AB, indicating their involvement in regulating biomass and nutritional quality.

DEGs and KEGG analyses revealed *SPS* gene was up‐regulated by 5.4‐fold at low and 6.3‐fold at high altitudes AB, which could enhance sucrose synthesis and carbon assimilation in *B. napus* . This could contribute to converting photosynthetics to stored sucrose, providing energy and materials for plant growth. *FBA1* gene was also up‐regulated by 4.4‐fold at high altitude, suggesting increased fructose bisphosphate aldolase activity, improving glycolysis and photosynthesis efficiency, especially in low‐temperature environments. *AGAL2* gene expression was elevated at high and reduced at low altitudes. This gene encodes an enzyme, which is crucial in high‐altitude conditions. It could potentially enhance metabolic pathways and stress responses in *B. napus* . These results imply that various genes modulate the biomass and nutritional quality of *B. napus* by regulating carbohydrate metabolism and glycolytic pathways, enhancing light energy utilization for carbon assimilation and energy production in low‐temperature environments.

## 4 Discussion

### 4.1 The Large Temperature Difference Between Day and Night at High Altitude Promotes the Growth of *B. napus* and Shortens the Picking Period

The length of picking period is directly related to the biomass and quality of *B. napus* for vegetable use. The altitude is an important natural factor (Eyre and Woodward 1988). The main factor affecting the length of picking period is temperature (Liu et al. 2005). The average annual temperature in the area of 1600 m altitude area was lower (12.70°C) than that in the area of 150 m altitude (18.85°C). The low temperature at high altitude prompted *B. napus* to show buds and blossoms earlier, significantly shortened the vernalization time of *B. napus* , and caused it to enter the reproductive growth stage earlier, leading to early bolting (Ma et al. 2021). The results showed that the picking period at 1600 m altitude was significantly shorter than that at 150 m altitude, with an average picking period of 15 d and 42 d. The large temperature difference between day and night at high altitude helped to reduce the respiratory consumption of *B. napus* during the night, which was favorable to the accumulation and storage of nutrients, and thus promoted the growth of *B. napus* and shortened the picking period (Xu et al. 2022).

### 4.2 Low Temperature at High Altitude Could Reduce Soil Microbial Activity, Which May Lead to Reduce the Absorption of Nitrogen, Phosphorus, and Kalium by Vegetable‐Use *B. napus*

Nitrogen, phosphorus, and kalium are a large number of elements required in *B. napus* growth process (Yang 2018). The results showed that the accumulation of N, P, and K of *B. napus* at 1600 m high altitude was lower than that at 150 m low altitude. Similarly, the N, P, and K contents of the soil at 1600 m altitude is lower than that at 150 m altitude. The study shows that with the increasing of altitude, the decreasing of temperature affects the soil microbial activity, and then affects the content of soil available N, P, and K elements (Li et al. 2018). The combined limiting effect of available N, P, and K elements in the soil led to the decreasing of N, P, and K elements absorption by *B. napus* (Yuan et al. 2017). The low temperature at 1600 m high altitude could reduce the contents of available N, P, and K elements in soil, which further reduced the absorption of N, P, and K by *B. napus* . Therefore, the contents of N, P, and K in *B. napus* at 1600 m high altitude were lower than that at 150 m low altitude.

### 4.3 The Higher Light Intensity at High Altitude Could Reduce the Biomass, Stem Length, and Stem Diameter of Vegetable‐Use *B. napus* and Increase the Number of Leaves of Vegetable‐Use *B. napus*

Stem diameter and fresh weight are important qualities of vegetable‐use *B. napus* . According to our results, the fresh weight and dry weight of *B. napus* showed the same trends with the increasing altitude. The fresh weight and dry weight of 1600 m altitude *B. napus* were significantly less than those of 150 m altitude *B. napus* . Other study also showed that the dry weight and fresh weight of the plants decreased gradually with the elevation increasing (Liu et al. 2020; Yang et al. 2022). With the increasing of altitude, the availability of resources in the above‐ground part of spruce decreased (Zhang et al. 2023b). At high altitude, spruce tended to invest in underground organs and produce a large number of fine roots to obtain nutrients, thus reducing the biomass of stems and leaves. This study showed that the fresh weight and dry weight of *B. napus* at low altitude were significantly higher than those at high altitude, suggesting that *B. napus* responded to the environment by reducing stem and leaf biomass allocation and actively adjusting biomass allocation at high altitude.

In agricultural production, the picked *B. napus* for vegetable use is mainly composed of three parts: stem, leaves, and buds. Stem diameter and stem length of *B. napus* are the direct index of biomass. As the altitude gradient increases, the light intensity increases and the ultraviolet ray increases (Pan et al. 2009). Plants mainly use visible light in long‐wave light for photosynthesis, and excessive ultraviolet rays will inhibit the elongation and growth of plant cells and make plants shorter (Ma et al. 2022). *B. napus* planted at a high altitude of 1600 m could be inhibited by ultraviolet rays, which limited the elongation and growth of *B. napus* . Therefore, the stem length and diameter of *B. napus* planted at 150 m low altitude are generally significantly higher than those planted at 1600 m altitude. This also indicated that *B. napus* would grow more upright and resist lodging at low altitude.

Leaves are one of the important organs for plants to receive light signals and perform photosynthesis (Yang, Zhan, and Zhang 2012). The number of leaves directly affects the light resources available to the leaves and determines the photosynthetic efficiency of the plants (Yang, Zhan, and Zhang 2012). Studies have shown that increased light in alpine environments led to smaller leaves. To make up for the adverse effects of shrinking photosynthetic area, plants will increase the number of leaves (Zhang et al. 2015). Our research shows that the numbers of leaves at 1600 m high altitude are significantly bigger than that at 150 m low altitude, the average number of leaves were 12 leaves and six leaves, respectively. This is consistent with previous research results (Yang, Zhan, and Zhang 2012; Li et al. 2017). This may be the reason that the light at high altitude is stronger, the leaf area of the *B. napus* would be reduced to protect the plant from the damage of high light intensity, and this could increase the number of *B. napus* leaves to improve the light‐capturing efficiency. Therefore, with the increase of altitude, the number of leaves in *B. napus* will increase.

### 4.4 The Soluble Sugar and Cellulose Content of Vegetable‐Use *B. napus* Is Reduced by Low Temperatures at High Altitudes

The stems and leaves of vegetable‐use *B. napus* are rich in ascorbic acid, soluble sugar, cellulose, protein, and other nutrients. The results showed that the soluble sugar contents of all *B. napus* varieties at high altitude were significantly lower than those at low altitude. Soluble sugars are mainly produced by photosynthesis, providing essential carbon sources and energy for plant growth and development (Durand et al. 2018). The right light at lower altitudes enhances the plant's photosynthetic capacity, which prompts the leaves to absorb more carbon and increase the soluble sugar content (Ntagkas et al. 2019). However, at higher altitudes, the decrease in temperature likely impairs photosynthetic efficiency, leading to reduced soluble sugar production (Chen 2015).

Furthermore, the variations in cellulose content among *B. napus* varieties at different altitudes are noteworthy. As the main component of cell wall, cellulose content has a certain correlation with the thickness of cell wall (Delmer and Haigler 2002). For vegetable use of *B. napus* , the cellulose content is generally required to be low. Interestingly, certain varieties such as Changxiangtai 502, Changxiangtai 603, Changxiangtai 701, Huayouza 655R, Hope 759, and Youtai 929 exhibited significantly higher cellulose content at 1600 m altitude compared to 150 m. The increase in cellulose may be attributed to an adaptive response to the low‐temperature conditions at high altitude. In high altitude conditions, certain varieties may accelerate the synthesis and accumulation of cellulose to strengthen the cell wall structure, which could provide additional support and protection for growing tissues (Guo et al. 2019). Conversely, other *B. napus* varieties showed lower cellulose content at higher altitudes. This decrease could be due to the inhibitory effect of low temperatures on plant growth, resulting in a reduced degree of cell wall fibrosis (Jinyu et al. 2023). These findings highlight the diverse responses of different *B. napus* varieties to environmental stresses, particularly in terms of cellulose content variation. In conclusion, the variations in soluble sugar and cellulose content observed in *B. napus* at different altitudes may be caused by the combined effects of temperature and light conditions.

### 4.5 High Light Intensity and Low Temperature at High Altitude Increase Ascorbic Acid and Protein Content in Vegetable‐Use *B. napus*

Studies have shown that ascorbic acid content of *B. napus* showed an overall significant positive correlation with altitude at 800–1800 m (Zhang et al. 2017). In the current study, at 1600 m altitude, it is noteworthy that the ascorbic acid contents of 23 varieties demonstrated notably elevated levels. This increase may be primarily caused by the influence of light (Paciolla et al. 2019), as plant exposure to light significantly boosts ascorbic acid content (Frohnmeyer and Staiger 2003). With increasing altitude, the intensity of light, potentially enhancing the photosynthetic efficiency and certain physiological processes in *B. napus* , ultimately leading to the accumulation of ascorbic acid (Cheng, Wang, and Meng 2012). The intense UV‐B radiation and low temperatures at high altitude introduce oxidative stress (Dong et al. 2021), which prompts the accumulation of ascorbic acid as a means to mitigate the harmful effects of ROS (Wang, Zhao, et al. 2018). However, it is noteworthy that some varieties like ZYCT03, Fenglv1, Huayouza655R, and Yuhua1 demonstrated lower ascorbic acid content at 1600 m compared to 150 m, possibly indicating their reduced adaptation in high‐altitude environments.

The protein content is another important factor that reflects the nutritional value of vegetables (Li 2016). According to the results, the protein content of *B. napus* at 1600 m altitude is generally more than the protein content of *B. napus* at 150 m altitude. This enhancement is linked to environmental factors such as sunshine and average daily temperature, where low‐temperature conditions favor protein formation (Jinyu et al. 2023). The increased protein content at higher altitude likely serves to raise the osmotic pressure of cells, thereby helping *B. napus* mitigate the adverse effects of the colder environment and ultimately exhibiting a higher nutritional value.

### 4.6 Low Temperatures at High Altitudes Can Induce Early Vernalization in Vegetable‐Use *B. napus* , Leading to Premature Reproductive Growth

To infer the growth status of *B. napus* at different altitudes, observations of SAM tissue were made. The results showed that the cells of the SAM changed from small to large and from tight to loose during the bolting. The transition of plants from vegetative growth to reproductive growth is influenced by various environmental factors, including photoperiod and temperature (Verocai et al. 2022; Luo et al. 2022). Some plants flower earlier when exposed to extended periods of cold temperatures, a process known as vernalization (Xu and Chong 2018). Simultaneously increasing the duration of vernalization and decreasing the vernalization temperature resulted in faster bolting and flowering (Woodhouse et al. 2021; Yow et al. 2023). The study revealed that in comparison to *B. napus* grown at low altitude, the cells in the meristematic tissues of those cultivated at high altitude exhibited greater size and reduced compactness. This difference may be caused by the low temperatures at high altitude, which induce early reproductive growth in *B. napus* . Consequently, this early growth leads to a shorter picking period.

### 4.7 Regulation of Key Genes of Metabolic Pathways in Vegetable‐Use *B. napus* by High Altitude and Bolting

Transcriptome analysis revealed that differential expression of *SPS, SUS, FBA1*, and *AGAL2* genes at different elevations and growth periods affected certain metabolic pathways and metabolite synthesis in vegetable *B. napus* . Sucrose is an important class of carbohydrates whose metabolism is closely related to plant growth and development and resistance to stress (Yow et al. 2023). *SUS*, *SPS*, and *INV* are the three key enzymes involved in sucrose metabolism (Duan et al. 2021). It is directly involved in the synthesis and distribution of sucrose and starch in plants, regulating the synthesis of sucrose. In agricultural practices, elucidating the impact of the *SPS* gene on the growth and flowering of *B. napus* is crucial for the development of varieties that can adapt to varying altitude conditions. *SPS* has been identified as playing an important role in floral development (Baxter et al. 2003). It was demonstrated that plants overexpressing the *SPS* gene improved sucrose synthesis and carbon assimilation, resulting in earlier and more flowering than wild‐type plants (Baxter et al. 2003). It was found that the *SPS* gene was up‐regulated about 6.3‐fold at high altitude after the bolting compared with BB. This up‐regulation of the *SPS* gene not only has implications for the growth cycle of *B. napus* but also extends to its yield and quality. Hence, in the breeding process, selecting varieties with optimized *SPS* gene expression could potentially enhance the adaptability and productivity of *B. napus* in high‐altitude regions.

*SUS* catalyzes the reversible conversion of sucrose and UDP to UDP‐glucose and fructose. *SUS* is believed to participate in cell wall biosynthesis by directly supplying UDP‐glucose to cellulose synthase (Haigler et al. 2001). Cellulose plays a crucial role in the development of floral organs (Cruz‐Valderrama et al. 2021). Reduced levels of synthetic cellulose result in a significant decrease in cellulose deposition on the pollen tube wall, leading to disorganization of the pollen tube wall layers and inhibition of floral organ development (Wang et al. 2011; Persson et al. 2007). *SUS* plays an important role in regulating carbon allocation to various sink tissues or organs. *CsSUS4* in cucumber is mainly expressed in sink organs, especially in flowers, fruits, and roots (Fan et al. 2019). *SUS* transgenic plants also result in accelerated plant growth and early entry into the reproductive period (Xu and Joshi 2010). The present study found that *SUS* was significantly up‐regulated in AB‐H and down‐regulated in BB‐H. This suggests that *SUS* can promote the synthesis of UDP‐glucose in *B. napus* to promote the development of floral organs and accelerate the reproductive period of *B. napus* .

Fructose‐1,6‐bisphosphate aldolase (FBA) is a versatile metabolic enzyme involved in multiple important processes of glycolysis, gluconeogenesis, and Calvin cycle (Zhao et al. 2019). Decreased FBA activity inhibits photosynthesis and alters carbon partitioning in potatos, whereas increased FBA activity promotes CO_2_ fixation and enhances growth and photosynthesis in tobacco (Haake et al. 1998; Uematsu et al. 2012). Also, FBA is involved in plant response to various environmental stimuli such as salinity, drought, anaerobic stress, abnormal temperature, and light acclimatization (Zhao et al. 2019). FBA acts as a key enzyme in glycolytic metabolism, providing precursors for amino acid and fatty acid synthesis (Haake et al. 1998). It has been shown that *FBAs* show the highest expression level in seed tissues, which can induce rapid seed embryonic growth, synthesize oil substances, and accumulate a large amount of protein content (Ma et al. 2008), so that the protein content of *B. napus* grown at high altitude is higher than that at low altitude. Research has found that low‐temperature stress could induce the expression of the genes *SIFBA4* and *SIFBA7*, and plants overexpressing *SIFBA4* and *SIFBA7* had a higher FBA activity and a smaller reduction in their photosynthetic rate, which significantly improved the tolerance of transgenic tomato plants to low‐temperature stress (Cai 2018). Overexpression of *FBA1* stimulates the regeneration of ribulose 1,5‐bisphosphate and promotes CO_2_ fixation, thereby increasing the rate of photosynthesis and biomass production in transgenic tobacco (Uematsu et al. 2012). In the context of agriculture particularly in high‐altitude regions with significant diurnal temperature variations and prolonged sunshine, these factors could potentially augment photosynthesis in *B. napus*. In this study, the expression of *FBA1* gene was up‐regulated in *B. napus* grown at high altitude to adapt to low‐temperature stress, and the gene may increase the protein content of *B. napus* by participating in the glycolytic pathway and the Calvin cycle. Research findings cottonseed sugar family oligosaccharides (RFOs) accumulated in cucumber leaves after low‐temperature stress and acted as osmoprotectants to alleviate stress injury, while α‐galactosidase was the key enzyme catalyzing the breakdown of RFOs (Man 2019). α‐galactosidase helped plants resist low‐temperature injury by participating in the metabolic process of oligosaccharides such as RFOs. Based on the aforementioned findings, it is evident that the *FBA1* gene possesses significant potential in agricultural applications, particularly in altitude‐specific breeding programs. In the future, the role of *FBA1* in enhancing photosynthesis and biomass production under varying temperature conditions can be harnessed to develop crops with improved stress tolerance and higher yields. This study revealed that *SPS*, *SUS*, *FBA1*, and *AGAL2* genes are key regulators of glycolytic and sucrose metabolism in *B. napus* , significantly influencing the plant's morphology, nutritional quality, and biomass accumulation across various altitudes and growth periods via modulation of metabolic networks.

### 4.8 Transcriptome Analysis, Microscopic Observation, and Physiological Indicators Together Reveal the Impact of High Altitude on Different Varieties of *B. napus*

The high‐altitude environment exerts multifaceted impacts on *B. napus* . At the molecular level, *B. napus* responds to environmental stresses by modulating the expression of specific genes, such as *FBA1* and *SUS*. Under high‐altitude conditions, the upregulation of the *FBA1* gene may contribute to increased protein content in *B. napus* . Additionally, the upregulated expression of the *SUS* gene could potentially enhance cellulose synthesis. The results of physiological indicators such as cellulose and protein content are consistent with the transcriptome analysis. In terms of cellular structure, the enlarged cell gaps in the *B. napus* SAM at high altitudes may indicate certain alterations in cell wall structure. Cellulose, a crucial component of the cell wall, is essential for maintaining cell shape and structural stability. However, the decrease in cellulose content was observed in most *B. napus* grown at high altitudes. The effect of low‐temperature stress on cellulose synthesis could result in a more relaxed cell wall structure and wider cell gaps. Together, these changes demonstrate the adaptive adjustments made by *B. napus* in high‐altitude environment.

### 4.9 *Brassica napus* in High Altitude: Adaptation Insights for Global Crop Production and Climate Resilience

As climatic conditions continue to change, the significance of high‐altitude areas in agriculture is increasingly prominent (Irshad et al. 2024). *B. napus* , a vital oilseed crop, should be studied for its adaptability in high‐altitude environments. These studies could not only provide a scientific basis for cultivating stress‐resistant, high‐yielding, and high‐quality crop varieties but also guide the adjustment and optimization of the global crop production system (Yang et al. 2021b). Furthermore, these findings underscore the importance of gaining a deeper understanding of crop's physiological and molecular responses to environmental changes. This is crucial for formulating effective strategies to adapt to climate change and ensuring global food security. Therefore, future research should continue to focus on the adaptive mechanisms of crops to extreme environments and how to utilize these mechanisms to enhance crop stress resistance and production efficiency.

### 4.10 Physiological Responses and Agricultural Strategies of *Brassica napus* at High Altitudes

The current study of 27 *Brassica napus* varieties across high and low altitudes has revealed a decrease in biomass with increasing altitude, accompanied by an elevation in ascorbic acid and protein content. These findings provide significant implications for the cultivation of *B. napus* in high‐altitude regions. Based on these research outcomes, we propose the following suggestions for agriculture in such environments: Firstly, breeders can capitalize on these discoveries to selectively cultivate *B. napus* varieties that are adapted to higher altitudes while preserving their nutritional value. Secondly, given the notable influence of altitude on the morphological and physiological traits of *B. napus* , we recommend that farmers tailor their planting density and fertilizer application rates to the specific conditions, thereby optimizing crop yields in these challenging environments. Lastly, the transcriptome analysis has uncovered the upregulated expression of certain genes in high‐altitude settings, presenting valuable opportunities to bolster the adaptability of rapeseed to these regions through genetic engineering strategies. Overall, this study serves as a valuable guide for high‐altitude rapeseed cultivar selection, agricultural practice adjustments, and the development of novel cultivar strategies.

## 5 Conclusion

Biomass and quality of vegetable *B. napus* were significantly affected by different altitudes. The biomass of *B. napus* was lower at high altitude. In terms of quality parameters, soluble sugar and fiber contents of *B. napus* at high altitude were lower, while ascorbic acid and protein contents of *B. napus* at high altitude were higher. In addition, elevation increasing was accompanied by a shorter picking period, as well as a significant decrease in fresh weight, dry weight, stem length, and stem thickness, while the number of leaves increased significantly. In terms of nutrient accumulation, N, P, and K accumulation in *B. napus* was lower at high altitude than at low altitude. It is noteworthy that Huayouza 655R showed significant inter‐altitude differences in all of the above indexes. Compared with pre‐bolting, paraffin sections of SAM AB showed morphological characteristics of increased cell volume and looser arrangement. Transcriptome analysis showed that genes such as *SPS*, *SUS*, *FBA1*, and *AGAL2* were significantly up‐regulated and expressed in AB‐L, AB‐H, and BB‐H. This study has deepened the understanding of high altitude cultivation affecting biomass and quality in *B. napus* , provided a scientific basis for trait regulation in breeding practice, and promoted the optimization process of genetic improvement and agricultural production in *B. napus* .

## Author Contributions

**Zongji Zhang:** funding acquisition (equal), investigation (equal), resources (equal), writing – original draft (equal). **Xionghua Li:** data curation (equal), investigation (equal), writing – original draft (equal). **Ri Ming:** investigation (equal), resources (equal), visualization (equal), writing – original draft (equal). **Yingying Lu:** data curation (equal), validation (equal), visualization (equal). **Qinwen Lin:** data curation (equal), investigation (equal). **Yafei Yang:** formal analysis (equal), investigation (equal). **Jialin Liao:** formal analysis (equal), software (equal), validation (equal). **Yunjuan Li:** investigation (equal), resources (equal), validation (equal), visualization (equal). **Lingli Mao:** investigation (equal), resources (equal). **Yang Huang:** funding acquisition (equal), software (equal), supervision (equal), writing – review and editing (equal). **Li Zhong:** formal analysis (equal), funding acquisition (equal), investigation (equal), supervision (equal), writing – review and editing (equal). **Yu Liang:** supervision (lead), writing – review and editing (lead).

## Ethics Statement

The authors have nothing to report.

## Consent

The authors have nothing to report.

## Conflicts of Interest

The authors declare no conflicts of interest.

## Supporting information


**Figure S1.** Growth patterns of *B. napus* grown at 1600 m altitude.

**Table S1.** *Brassica napus* picking period at different altitudes.

**Table S2.** Nutrients of different altitudes of *B. napus* .

**Table S3.** Soil nitrogen, phosphorus, and kalium contents.

**Table S4.** Soluble sugar content of *Brassica napus* at different altitudes.

**Table S5.** Cellulose content of *Brassica napus* at different altitudes.

**Table S6.** Ascorbic acid content of *Brassica napus* at different altitudes.

**Table S7.** Protein content of *Brassica napus* at different altitudes.

**Table S8.** Differentially expressed genes discussed in AB‐L and BB‐L.

**Table S9.** Differentially expressed genes discussed in AB‐H and BB‐H.

**Table S10.** Differentially expressed genes discussed in BB‐H and BB‐L.

**Table S11.** Differentially expressed genes discussed in AB‐H and AB‐L.

## Data Availability

The raw sequencing data have been deposited to the NCBI (https://submit.ncbi.nlm.nih.gov) with Sequence Read Archive (SRA) accession No. PRJNA1094408.
