# Supplementary material for: Microscopical Observation and Transcriptome Analysis Reveal the Effects of High‐Altitude Ecosystem in the Qualities of Different Genetic Varieties Brassica napus Resources
Source: Ecol Evol. 2024 Nov 21;14(11):e70616. doi: 10.1002/ece3.70616 (PMC11582087; doi:10.1002/ece3.70616)
Supplement: Supplementary file 1 — Figure S1. Growth patterns of B. napus grown at 1600 m altitude. [file ECE3-14-e70616-s004.pdf]

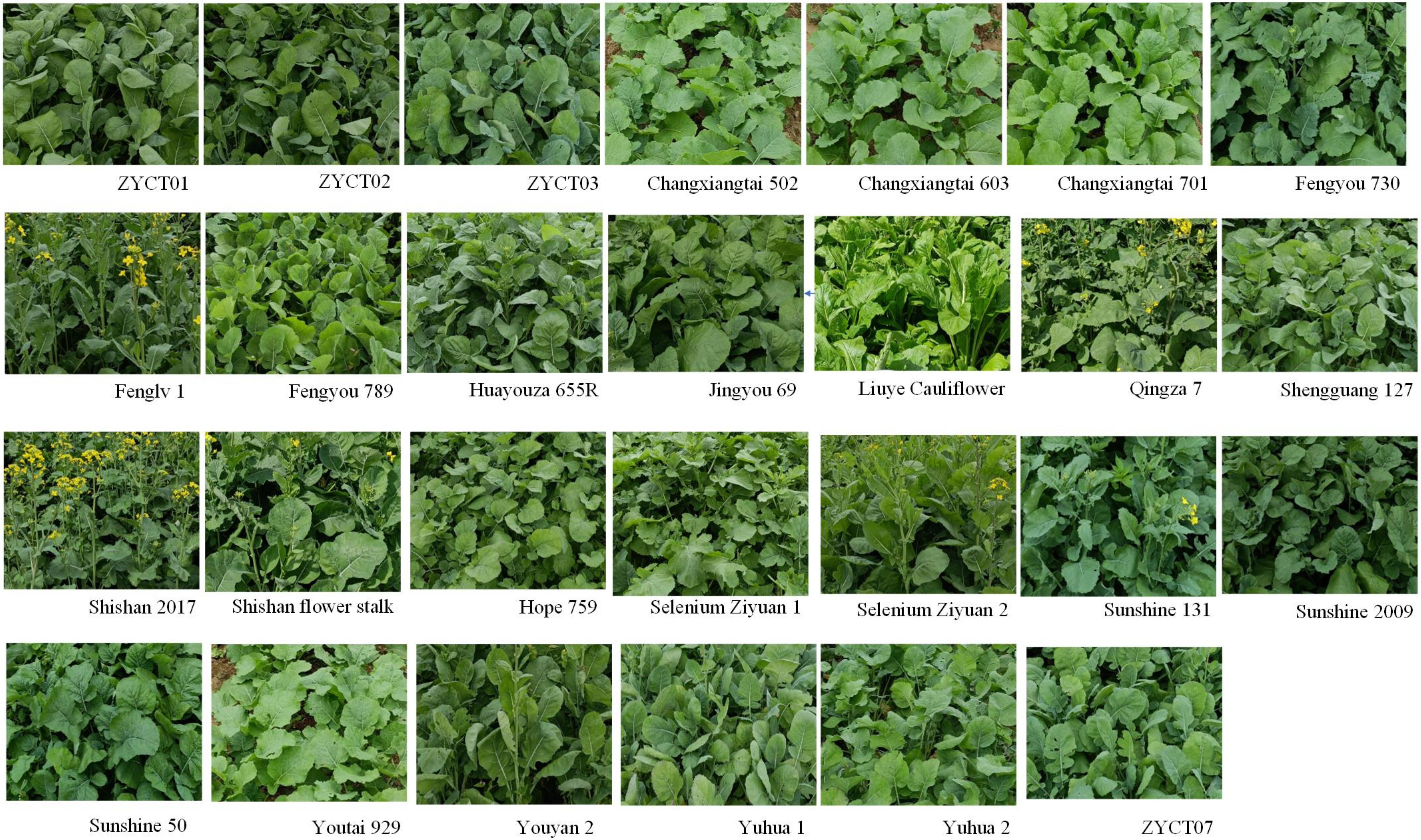
ZYCT01
ZYCT02
ZYCT03
Changxiangtai 502
Changxiangtai 603
Changxiangtai 701
Fengyou 730
Fenglv 1
Fengyou 789
Huayouza 655R
Jingyou 69
Liuye Cauliflower
Qingza 7
Shengguang 127
Shishan 2017
Shishan flower stalk
Hope 759
Selenium Ziyuan 1
Selenium Ziyuan 2
Sunshine 131
Sunshine 2009
Sunshine 50
Youtai 929
Youyan 2
Yuhua 1
Yuhua 2
ZYCT07
